# Evaluation of horizontal gene transfer risk between the Mediterranean fruit fly *Ceratitis capitata* (Tephritidae) and its parasitoid *Fopius ceratitivorus* (Braconidae)

**DOI:** 10.1371/journal.pone.0207999

**Published:** 2018-12-04

**Authors:** Edwin Ramírez-Santos, Pedro Rendón, Kostas Bourtzis, Marc F. Schetelig, Carlos Cáceres, Asya Targovska, Tanja Rehling, Griselda K. Guillén-Navarro, Lorena Ruiz-Montoya, Jorge Toledo, Pablo Liedo

**Affiliations:** 1 El Colegio de la Frontera Sur (ECOSUR), Carretera Antiguo Aeropuerto Km. 2.5, Tapachula, Chiapas, Mexico; 2 Laboratorio El Pino, Programa MOSCAMED, Km. 47.5 carretera a El Salvador, Parque Nacional Laguna El Pino, Santa Rosa, Guatemala; 3 IAEA/TC-LAC, Guatemala City, Guatemala; 4 Insect Pest Control Laboratory, Joint FAO/IAEA Division of Nuclear Techniques in Food and Agriculture, Seibersdorf, Austria; 5 Institute for Insect Biotechnology, Justus-Liebig-University Gießen, Gießen, Germany; 6 El Colegio de la Frontera Sur (ECOSUR), Carretera Panamericana y Periférico Sur s/n, San Cristóbal de Las Casas, Chiapas, Mexico; University of Thessaly School of Agricultural Sciences, GREECE

## Abstract

The transgenic strain of the Mediterranean fruit fly (medfly), *Ceratitis capitata* (Wied.) VIENNA 8 1260, developed from the classical genetic sexing strain VIENNA 8, has two molecular markers that exhibit red fluorescence in the body and green fluorescence in testicles and sperm. These traits offer a precise tool to discriminate between mass-reared sterile males and wild fertile males, and they could potentially increase the effectiveness of control programs for this pest. To assess the risk of horizontal transfer of the fluorescence transgenes in natural ecosystems, we used the VIENNA 8 1260 strain and the medfly parasitoid *Fopius ceratitivorus*. The fluorescence signal and the inheritance of the fluorescence gene markers were monitored for over 16 generations (about two years) in both species using fluorescence microscopy and a PCR-based assay. The PCR analysis was performed in four independent laboratories. Both fluorescence microscopy and PCR analysis indicated that no horizontal gene transfer of the DsRed transgene occurred during 16 generations of medfly parasitoid rearing under experimental conditions.

## Introduction

The order Diptera, which includes mosquitoes and fruit flies, harbours several invasive insect species that can have a negative impact on agriculture and human health [1]. The distribution of invasive mosquito and fruit fly species is favored by factors such as global climate change [2], growing international trade [3] and increasingly vulnerable zones that promote pest establishment [4–6]. Countries and regional organizations establish control programs to eradicate them or prevent their establishment. A challenge for these programs is the identification of highly effective control tools with low cost and environmental impact. Such is the case of the Sterile Insect Technique (SIT) that has been essential for the population control of several species of fruit flies under different programs of Area-Wide Integrated Pest Management (AW-IPM) around the world [7].

One of the advances in insect population control has been the development and use of genetically improved strains of insects created by classical genetic techniques and, more recently, genetic engineering. By the end of the past century, classical genetic sexing strains were developed to allow the separation of *Ceratitis capitata* males and females [8]. To get a male-only population, eggs from the GSS strain are heat treated for 24 h at 34°C which leads to the elimination of all females at the embryonic stage [9].This breakthrough significantly improved the population control of this pest [10–12]. The genetic sexing strain VIENNA 8 allows the production of a male-only population upon heat shock at the embryonic stage. The system is based on a reciprocal translocation between the chromosome that determines the male sex (Y) and an autosomal chromosome (5) that carries the wild-type alleles of two genetic markers, the temperature sensitive lethal (*tsl*) gene and the white pupae gene (*wp*), used to build up the genetic sexing mechanism. This translocation of the *wp* and the *tsl* wild type alleles from the autosome 5 to the Y chromosome, namely the translocation 101, renders male pupae brown and resistant to thermal treatment.

Recombinant DNA technologies have been used to design and develop transgenic fruit fly strains, with desirable traits for mass rearing and field operations, enhancing their ability to control the target pests [13, 14]. An example is the transgenic, sperm-marked strain VIENNA 8 1260 which expresses a red fluorescent protein on the pupae and body of the adults, and a green fluorescent protein in the male testicles and sperm [15]. This strain could potentially improve the monitoring process during an SIT program by accurately discriminating mass-reared, sterilized, and released (fluorescent) males from wild (non-fluorescent) males [16]. In addition, it may offer an alternative to the current marking procedure which is based on the dying of pupae with fluorescent powder which gets sequestered in adults during their emergence [17, 18]. This technique is reliable and cost-effective but false diagnosis of insects due to the loss of the fluorescent powder may have significant impact on the cost of a release program [19].

However, the use of such novel strains may be hindered by concerns about the risks related to their potential impact on the environment and natural ecosystems or to the social perception on the use of transgenic organisms [20]. The assessment of the potential ecological impact of transgenic organisms is challenging due to the dynamics of each species either at the local population or metapopulation level [21–23]. The transgenesis may have a fitness cost for the species genetically modified [24] which is not desirable if these strains will be used as a biological control agent, such as the VIENNA 8 1260 potential use for the SIT-Area Wide control of the medfly. So, the use of transgenic insects under field conditions should be done under a strict regulatory framework taking into consideration guidelines as well as technical and biological warranties as the ones reported by the North American Plant Protection Organization, NAPPO [25] and the European Food Safety Authority, EFSA [26] in order to eliminate, if possible, the risk of spreading transgenes in the populations of the targeted pest species. Towards this goal, the performance and quality parameters of the transgenic VIENNA 8 1260 under mass rearing conditions were recently evaluated [27–28] and a full description of the transgene composition and how this strain has been generated is available [29]. In addition, the irradiation dose, that ensures optimal mating performance of the sterile males and level of induced sterility on the natural population in the field and at the same time minimizes or eliminates the risk of transferring the fluorescence transgene to the wild fly populations, was determined.

An additional concern with respect to the use of transgenic insects in open field releases is the potential horizontal (between species) transmission of transgenes. The use of irradiation to fully sterilize insects for SIT applications ensures that there will be no vertical transmission of any transgene in nature. However, this does not prevent their potential horizontal transmission through, for example, transposable elements, symbionts, parasites or viruses [29–32]. The horizontal transfer of genes has been documented in many prokaryotic and eukaryotic species, as well as within and between kingdoms, and it’s particularly pronounced in insects in respect of the transfer of transposable elements as well as genes or even entire genomes of symbiotic bacteria [33–41]. Although engineering the transposons used in the germ line transformation of insects minimizes their instability in transgenic insects [42], the risk of horizontal transfer of the transgenes still exists since such transfers may be mediated by other natural mechanisms. Taken together, risk assessment studies are needed to *quantify*, if possible, the likelihood that such an event may occur during the time frame of a typical medfly population control program, lasting from a few months up to 10 years [43]. The results of this study should only be interpreted in terms of SIT programs, but not in terms of a general evolutionary risk of HGT in insects, which has been reported in the literature and might occur over a much longer time frame [44].

*Fopius ceratitivorus* Wharton (Hymenoptera: Braconidae) was selected for this study, because it is a promising egg-larval parasitoid for the population control of medfly, thus providing the opportunity to monitor by means of PCR, the potential HGT of the VIENNA 8 1260 medfly fluorescence transgene to the parasitoids. This parasitoid was originally reported from fruit flies collected in coffee berries from Kenya [45], which is part of the African region where both coffee and the medfly are originated. A small colony of *F*. *ceratitivorus* has been established at the San Miguel Petapa laboratory from parasitoids collected in Kenyan coffee plantations and introduced to Guatemala through USDA-APHIS following proper quarantine and biosafety protocols [46].

*Fopius ceratitivorus* is currently being mass-reared in Guatemala [47] and tested in medfly-infested areas. There are ongoing efforts to mass-rear this species at Hawaii [48]. In Guatemala, coffee (*Coffea arabica* L.) is the main host plant for *C*. *capitata* (Wied.) and its biology, worldwide distribution, host plant preferences and genetic variability has been extensively documented [49–58]. The medfly-parasitoid interaction provides an ideal scenario to test the HGT risk between these two species, given the nature of the parasitoid’s life cycle, in close contact with the medfly host. The presence of the fluorescent transgene in VIENNA 8 1260 medflies adds the possibility of detecting a potential HGT by the detection of the DsRed transgene through epifluorescence microscopy of the parasitoids, that would complement the PCR analyses and allows for a quick monitoring of HGT in adult parasitoids.

## Materials and methods

In the present study, the parasitoid *F*. *ceratitivorus* was reared on the transgenic VIENNA 8 1260 strain for 16 generations, a time period representing about 2 years, with the purpose of detecting the potential horizontal transfer of the transgenes from the fly host to the parasitoid by means of fluorescent microscopy and PCR analysis.

The parasitoid *Fopius ceratitivorus* Wharton is routinely reared at San Miguel Petapa (SMP) laboratory, on a bisexual strain of *C*. *capitata* -Tolimán/99 as a host following a validated methodology [47]. For the purpose of the present study, the parasitoids were reared on the transgenic *Ceratitis capitata* strain VIENNA 8 1260 for 16 generations (G_1_ to G_16_). In each generation, VIENNA 8 1260 females laid eggs in apple (*Malus domestica* Borkh) fruits. These fruits were then exposed to the parasitoid *F*. *ceratitivorus*, whose adults were recovered afterwards and a new cycle started. The VIENNA 8 1260 females came from a small colony (generation 36) following the filter rearing system, FRS [59], reared at the quarantine area of the SMP laboratory, located at 15 km southeast of Guatemala city (N 14° 29´ 2”, W 90° 36´ 53”).

### Infestation of apple fruits with VIENNA 8 1260 eggs

Under controlled environmental conditions, 3000 males and 3000 females of the VIENNA 8 1260 strain were kept inside 30 × 30 × 30 cm square cages (SC) with 0.5 mesh plastic on their six sides. The flies were fed *ad libitum* with a mixture of 3:1 sucrose: hydrolyzed yeast and water contained in 200 ml plastic container (8.2 cm H × 6.5 cm in diameter) covered with a cotton plug. To facilitate fruit oviposition by the medfly and later its parasitoids, the apples were prepared by puncturing them with a stainless-steel pin, thus creating approximately 1500 punctures per fruit. After a mating period of 3 days, the apples (4–6 per cage) were exposed to the VIENNA 8 1260 females as an oviposition substrate. The exposure was repeated three times per cage and, in each case, two apples were left inside the cage for 24 h. All the infested apples were then kept for 24 h in a room at 24°C and 65% relative humidity to promote the embryonic development of the VIENNA 8 1260 eggs. This procedure was repeated in each generation.

### Parasitization of the VIENNA 8 1260 eggs by *F*. *ceratitivorus*

A total of 2000 males and 2000 females of the *F*. *ceratitivorus* parasitoids kept inside 30×30×30 cm^3^ cubic plexiglass cages were fed with honey and water. The parasitoids were kept for 7 days, until they reached sexual maturity and mated. Then, the apples infested with VIENNA 8 1260 eggs were introduced for 24 h in the cages with the parasitoids, thus allowing the colonization of the VIENNA 8 1260 eggs by them. After the parasitization period was ended, each apple was cut into four parts and put on artificial standard larval diet to feed the parasitized VIENNA 8 1260 larvae for 6 days. This diet is a modification of the standard Seibersdorf diet described by Braga et al. (2006) [60]. The VIENNA 8 1260 larvae were collected and pupated. The VIENNA 8 1260 adults (males and females) emerged at day 12 after pupation, and the adult parasitoids (males and females) three days later.

The adult parasitoids recovered in each generation were introduced again in 30×30×30 cm^3^ cubic plexiglass cages and the parasitization procedure of the VIENNA 8 1260 eggs was repeated for 16 generations. In each generation, six replicates samples of 6–10 randomly selected adult parasitoids of each sex were preserved in absolute ethanol inside 1.5 ml Eppendorf vials (6 vials per sex per generation) for DNA analysis.

### PCR analysis of the parasitoid samples reared on VIENNA 8 1260 flies

To evaluate the potential horizontal transfer of DNA sequences of the fluorescence transgene from the VIENNA 8 1260 flies to its parasitoid, DNA from *F*. *ceratitivorus* parasitoids reared on the transgenic flies was extracted and amplified by PCR with 1260DsRed-F (AGCTGGACATCACCTCCCACAACG) and 1260DsRed-R (GTACTGGAACTGGGGG GACAG) (Macrogen Inc., Rockville, USA) primers specific for the fluorescence gene markers present in VIENNA 8 1260 [61]. The PCR analysis was conducted on parasitoids collected from generations G_0_, G_11_, G_12_ and G_14_ at the Agroecological and Environmental Biotechnology Laboratory (LaBTAA) from El Colegio de la Frontera Sur (ECOSUR), Tapachula, Chiapas, Mexico. Samples of parasitoids from generations G_0_ to G_16_ were also analyzed at three additional laboratories: a) Genetic Laboratory of ECOSUR, Unidad San Cristóbal (México); b) the Insect Pest Control Laboratory of the Joint FAO/IAEA Division of Nuclear Techniques in Food and Agriculture in Seibersdorf, Austria, and c) the Institute for Insect Biotechnology at the Justus-Liebig-University Gießen in Gießen, Germany.

DNA was extracted, individually, from all male and female parasitoids sampled from generations G_0_, G_11_, G_12_, G_14_, and G_16_. For the PCR amplification, a 1.0 μl of the extracted DNA and a 2.0 μl from a 1:10 dilution was analyzed using specific primers 1260DsRed-F and 1260DsRed-R for the detection of the DsRed fluorescence transgene. The PCR parameters were: 95°C for 5 min; 30 cycles of 95°C for 30 sec, 56.8°C for 30 sec, 72°C for 1 min; and final extension 72°C for 5 min.

The PCR was analyzed on 1% agarose gels loaded with 10 μl out of the 20 μl of the PCR product. The size of the DNA bands was estimated by observing them under UV light and comparing their migration distance with that of the molecular weight markers coming from a 1 kb DNA ladder (O’ Gene Ruler 1Kb Plus DNA Ladder, Thermo Scientific, No. Cat #SM1343). As positive control, an amplicon of 700 bp of the DsRed sequence from a VIENNA 8 1260 fluorescent adult was generated, while the negative control was DNA from a *F*. *ceratitivorus* adult parasitoid reared on a wild type strain of *C*. *capitata*, with no contact to the VIENNA 8 1260 strain.

To verify the quality of the DNA extraction and the reliability of the PCR results, another PCR was performed with a 1.0 μl subsample of the DNA previously extracted from the parasitoids samples and 2.0 μl of their 1:10 dilution using the NS1-GCFung primers [62] directed at the 18S eukaryotic ribosomal unit marker, and the following PCR conditions: 95°C for 5 min; 35 cycles of 95°C for 30 sec; 50°C for 40 sec; 72°C for 1 min; and 72°C for 5 min. A 10 μl subsample from the 20 μl PCR product was analyzed by electrophoresis on 1% agarose gels. DNA band sizes were estimated by comparison to a 1 kb ladder molecular weight marker (O’ Gene Ruler 1Kb Plus DNA Ladder, Thermo Scientific, No. Cat #SM1343). As positive control for DNA extraction, an amplicon of 500 bp of the 18S marker from a VIENNA 8–1260 fluorescent adult was generated; as the negative control was a reaction mixture with all the reagents used in the PCR processing except DNA.

### Fluorescence microscopy analysis and persistence of the fluorescent protein expression through time

Complementary to the PCR analysis, a fluorescence microscopy analysis of the parasitoids reared on VIENNA 8 1260 fluorescent flies was conducted from G_0_ to G_16_ (1200 male and 1200 female parasitoids per generation, for a total of 40,800) in order to verify their ability to express (or not) red or green fluorescence under UV light and specific filters: GFP Plus 510/20-nm band pass filter for green fluorescence and Texas Red with a 75-nm emission window (608 to 683-nm) and a 55-nm excitation passband (532 to 587-nm) for red fluorescence, with the aid of a Leica MZ-FL III stereoscope.

To test the persistence of the fluorescent protein expression though time, triplicate samples of the G_0_ males and females in 100 adult grids were kept under laboratory conditions (24°C and 65% RH) with no food or water until they died (about 6 days later) and the protein expression was verified weekly under the fluorescence microscope for up to 10 weeks after their death, which is about 10 times the lifespan of a field trap. A similar triplicate set of 100 male and 100 female adults in grids was also kept under field cage conditions, with a wide range of external environmental temperature and humidity (14–33°C, 40–100% RH) and analyzed with the same procedure.

## Results and discussion

**Fig 1:**
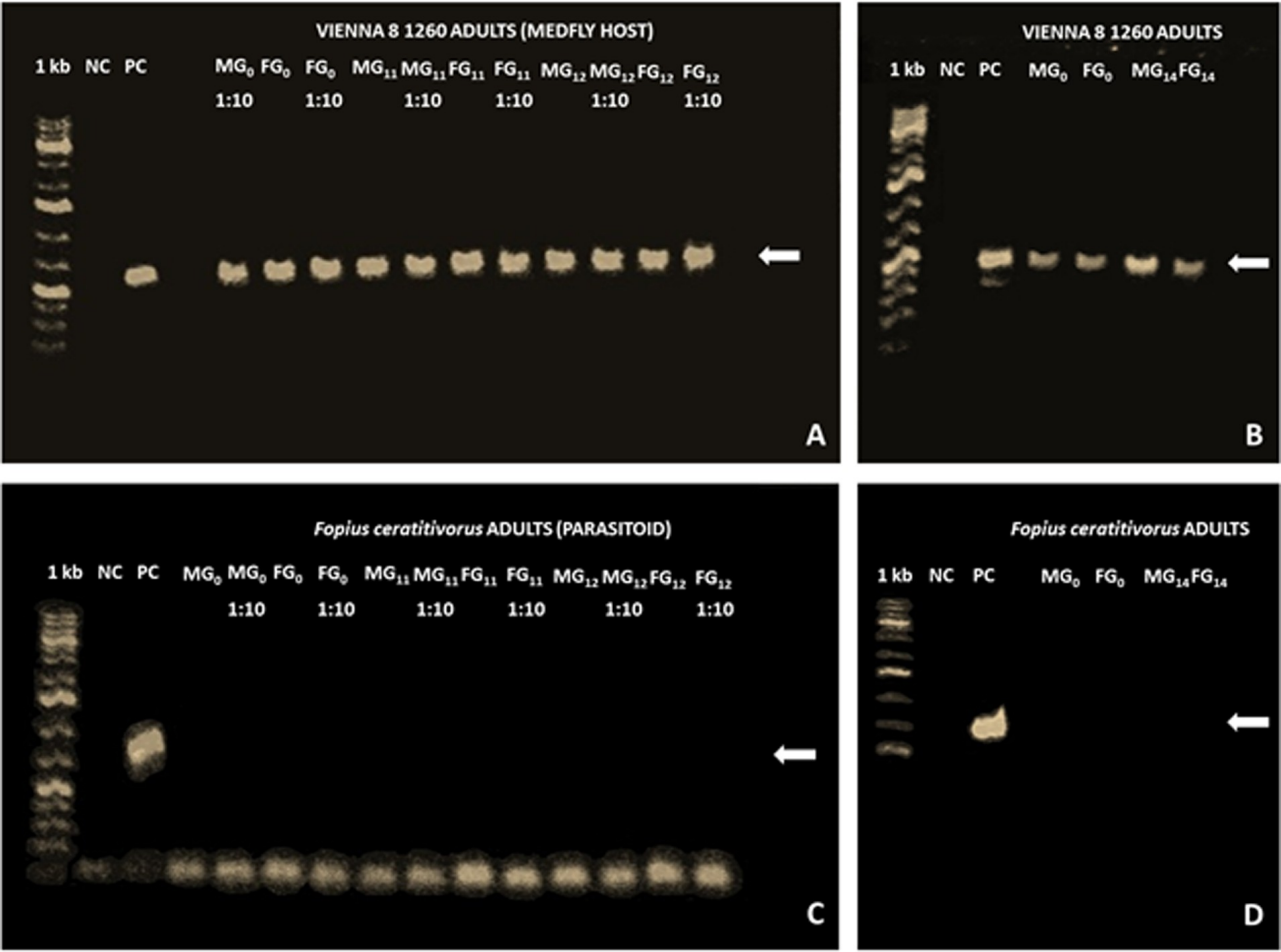
Examples of 1% agarose gel electrophoresis of PCRs specific for the DsRed transgene. (A) 1024 × 563 pixels and (B) 847 × 968 pixels pictures of PCR for VIENNA 8 1260 medfly host flies. (C) 702 × 767 pixels and (D) 847 × 968 pixels pictures of PCR for *F*. *ceratitivorus* parasitoids reared on VIENNA 8 1260. G_0_ parasitoids represent the initial generation without contact with VIENNA 8 1260. 1 kb = DNA ladder. NC = negative control. PC = positive control = 700 bp amplicon of the DsRed transgene. M = male. F = female. G_n_ = nth generation. 1:10 = 1:10 dilution of the DNA extraction sample. The white arrows to the right show the position of the DsRed fluorescence marker, present in all VIENNA 8 1260 adults and absent in all *F*. *ceratitivorus* adults.

PCR analysis performed at the Agroecological and Environmental Biotechnology laboratory (LaBTAA) of El Colegio de la Frontera Sur (ECOSUR), Unidad Tapachula (Mexico) indicated the presence of the DsRed fluorescence marker gene in all VIENNA 8 1260 adults analyzed at generations G_0_, G_11_, G_12_ (Fig 1A), G_14_ (Fig 1B) and G_16_. Male and female parasitoids from generations G_0_ (20♂+20♀), G_11_ (18♂+18♀), G_12_ (18♂+18♀) (Fig 1C), G_14_ (20♂+20♀) (Fig 1D) and G_16_ (18♂+18♀), reared on transgenic VIENNA 8 1260 host flies showed the absence of the marker corresponding to the DsRed transgene. All DNA samples were of high quality as was confirmed by the amplification of a fragment of the 18S eukaryotic ribosomal subunit gene at additional PCR analyses. No horizontal gene transfer of the DsRed transgene was detected for 16 generations (about 2 years) under the experimental conditions of this study. These results are consistent with the expected expression of fluorescence in the VIENNA 8 1260 males [15, 19, 27, 28] and they are not in conflict with the potential HGT between insects at an evolutionary level [42]. They only support the conclusion that such an event is unlikely to occur within the time frame of a medfly population control program [43], (probability < 1/40,800 or <0.000025, based on the number of individuals examined).

PCR results obtained from the other three laboratories testing the same G_0_ (18♂+18♀) and G_16_ (18♂+18♀) samples, are summarized in Table 1. In all independent assays, no amplification of the DsRed sequence was detected in the parasitoid samples tested, while a DsRed amplicon was amplified in the positive control. The fact that all different laboratories came up with the same results strengthen the validity of the results by lowering the chance of false negative results due to failures of the PCRs.

**Table 1:** PCR results on detecting the DsRed transgene in *F*. *ceratitivorus* parasitizing the transgenic *C*. *capitata* VIENNA 8 1260 strain. Sample = generations 0, 11, 12, 14, and 16. Microscopy = examination under a fluorescence stereoscope. 18S gene = PCR amplification of the 18S DNA. PCR = electrophoresis of the PCR amplification products in *F*. *ceratitivorus* using the 1260DsRed-F and 1260DsRed-R primers. PC = positive control VIENNA 8 1260. NC = negative control *F*. *ceratitivorus* reared on wild type strain (no contact with VIENNA 8 1260). Laboratories: (1) LaBTAA, Tapachula (Mexico), (2) Genetic Laboratory of ECOSUR, Unidad San Cristóbal (México), (3) FAO/IAEA Seibersdorf (Austria), (4) Institute for Insect Biotechnology, Gießen (Germany).

		18S gene		PCR pure		PCR 1:10 dilution		
Sample	Microscopy	*Fopius* ♂	*Fopius* ♀	*Fopius* ♂	*Fopius* ♀	*Fopius* ♂	*Fopius* ♀	Lab
G0	-	+	+	-	-	-	-	(1,2,3,4)
G11	-	+	+	-	-	-	-	(1)
G12	-	+	+	-	-	-	-	(1)
G14	-	+	+	-	-	-	-	(1)
G16	-	+	+	-	-	-	-	(1,2,3,4)
PC	+	+	+	+		+		
NC	-	-	-	-		-		

Fluorescence microscopy data are summarized in Table 1. Though this table only presents results for the same parasitoid generations analyzed by PCR, namely G_0_, G_11_, G_12_, G_14_ and G_16_, in fact no fluorescence was detected in the generation by generation analysis of 1200 males and 1200 females per generation of *F*. *ceratitivorus* from G_0_ to G_16_. The positive controls (VIENA 8 1260 male and female adults) showed 100% fluorescence expression for all transgenic adults parasitized at the same generations as the examined parasitoids. As an alternate way of determining a fully functional DsRed gene being horizontally transferred, the presence of the DsRed fluorescent marker in the parasitoids was checked. In an eradication program, millions or even billions of males are released in the field each week. If, for any reason (failures in the irradiation procedures, accidental releases of fertile males or intentional releases of transgenic lethality strains that have not been treated with radiation) they produce offspring, those might be colonized by the *Fopius* parasitoids.

Taken together, the data indicates that no horizontal gene transfer (HGT) of the DsRed fragment from the transgenic medfly host to the parasitoid genome was detected, under the experimental conditions of the study (rearing of the *F*. *ceratitivorus* on transgenic medflies for a period of 16 generations); we considered that this time period was adequate to assess the risk of HGT, taking into account that for eradications or management programs, this is a good representation.

Additionally, this study demonstrated that fluorescent protein is expressed in the VIENNA 8 1260 adults for as long as there is any soft tissues inside their exoskeletons, which was observed for at least 10 weeks after their death in all 100 male and 100 female grid samples kept under laboratory conditions. In a real world scenario, traps for monitoring are checked every week, so that this time period is about 10 times the lifespan of a field trap. The same results were obtained from field cage samples examined under the same protocol.

This study assesses the risk of horizontal transfer of the fluorescent transgene DsRed from the transgenic medfly strain VIENNA 8 1260 to *F*. *ceratitivorus*, during rearing of the parasitoid for 16 generations on its host. This time period represents about two years of repeated contact between the *F*. *ceratitivorus* parasitoids and the transgenic VIENNA 8 1260 host. Our analysis was based on fluorescence microscopy in order to see if the transgenic cassette has been transferred to the parasitoid in a way that maintained its functionality. However, no evidence of red fluorescence was detected in 8,867 parasitoids screened during sixteen generations of rearing, including males and females in a 1:1.37 ratio. In addition, we performed PCR analysis of 92 male and 92 female randomly selected parasitoids in five generations G_0_, G_11_, G_12_, G_14_, and G_16_ in order to see whether the functional transgene was horizontally transferred from the medfly VIENNA 8 1260 strain to the parasitoid. No fluorescent signal could be detected by fluorescence microscopy analysis. The results were again very clear in that no detection of the expected 500 bp long amplicon of the DsRed marker could be detected. Taken together, PCR and fluorescence microscopy analysis provided no evidence of HGT event, at least under the experimental conditions of the present study.

The recent use of transgenic organisms in health and agriculture has generated an intense debate between two main groups. One that opposes to the use of transgenic organisms, perceiving in their use the *possibility* of an horizontal transfer of genes and their potential impact in the natural ecosystems and human health, and a second one who favors their use that balance their benefits against the *probability* that such horizontal transfer may indeed occur. In the short term, it is such an extremely rare event, that it will unlikely play a role on the environment. However, it’s well known that HGT events (a) can take place even between members of different domains of life [63] and (b) have been commonly detected in insects being mediated by transposable elements, symbionts like *Wolbachia* and viruses, so risk assessment analysis is needed to address the concerns of releasing transgenic insects for field applications against insect pests and disease vectors.

## Conclusions

In the present study, we assessed the potential of HGT between the medfly and its parasitoid *F*. *ceratitivorus* during the typical time frame of an eradication program. We were unable to detect any HGT event. However, the data of this study should be read with caution since no detection does not necessarily mean that there have not been HGT events which we could not detect because: (a) a different part of the transgene cassette than that tried to detect by PCR was moved; (b) the transferred fragment carry mutations and this may explain the lack of fluorescence signal and PCR amplification; (c) it is practically impossible to check all individuals in every single generation and so, our PCR analysis was performed only in a limited number of insects and in just four out of sixteen generations and, last but not least, (d) nothing excludes the possibility that such an HGT event is so rare that we could not detect it in just 16 generations with that size of parasitoid population used in our experiment.

The persistence of the fluorescence expression in VIENNA 8 1260 adults for at least ten weeks under both laboratory and field cages conditions shows the stability of this fluorescent protein. Nevertheless, under different environmental conditions (higher humidity or extreme temperatures) this protein might degrade faster and further research needs to be conducted to test the durability of the protein for those conditions.
